# Helminth control in kennels: is the combination of milbemycin oxime and praziquantel a right choice?

**DOI:** 10.1186/s13071-015-0647-2

**Published:** 2015-01-17

**Authors:** Laura Rinaldi, Saverio Pennacchio, Vincenzo Musella, Maria Paola Maurelli, Francesco La Torre, Giuseppe Cringoli

**Affiliations:** Department of Veterinary Medicine and Animal Productions, University of Naples Federico II, CREMOPAR, Naples, Regione Campania Italy; Department of Health Sciences, University Magna Græcia, Catanzaro, Italy; Novartis Animal Health, Rome, Italy; Faculty of Veterinary Medicine, University of Teramo, Piazza A. Moro, 45, 64100 Teramo, Italy

**Keywords:** Kennels, Intestinal helminths, Cardio-pulmonary helminths, Control, Milbemax®, Drontal Plus Flavour®, Nemex® POP

## Abstract

**Background:**

Kennel dogs are at the high risk of infections with intestinal and extra-intestinal helminths. Therefore, regular parasitological surveillance, appropriate treatment strategies and high quality standard of hygiene are required to guarantee the health and welfare of kennel dogs. The aim of the present study was to evaluate the efficacy of helminth control in kennels using different broad-spectrum anthelmintics that are routinely used in canine veterinary practice. Particular attention was given to the field efficacy and ease-of-use of each product.

**Methods:**

The study was conducted in 3 public kennels in the Campania region (southern Italy). Eighteen boxes from each of the three kennels were selected for treatment based on faecal egg counts (FECs) at Day −30. The treatments were conducted using tablets containing combinations of: i) milbemycin oxime and praziquantel (Milbemax®); ii) pyrantel embonate, febantel and praziquantel (Drontal Plus Flavour®), and; iii) pyrantel pamoate, oxantel pamoate and praziquantel (Nemex® POP). All dogs were treated on Day 0 and sampled on Days 0, 7, 14 and 21 for copromicroscopic analyses. The FLOTAC dual technique on pooled samples was used with efficacy determined by reduction in FECs.

**Results:**

At Day −30 intestinal nematodes (hookworms, *Toxocara*, *Trichuris*) and cestodes (*Dypilidium caninum*) were found. Milbemax® showed 100% efficacy against all the helminths in all the kennels. Drontal Plus Flavour® was 100% effective against hookworms in all the kennels but gave lower efficacy against *T. canis* (range = 97.1-100%) and *T. vulpis* (range = 95.6-100%). Nemex® POP was also 100% effective against hookworms in all kennels but less effective against *T. canis* (range = 95.7-100%) and *T. vulpis* (range = 95.7-100%). All three drugs were 100% effective against *D.caninum*.

**Conclusions:**

Milbemax®was fully effective against all the helminthes and was palatable and ease-of-use for all the dogs. It is the optimum choice for the treatment and prevention of helminth infections in kennel dogs under field conditions.

## Background

Infections of dogs with helminths are often neglected, i.e. “not treated with proper attention” with respect to their diagnosis, surveillance and control. In the last few decades, especially among clinicians and practitioners, there was a misconception that helminths infecting pets, especially intestinal nematodes and cestodes, do not warrant much attention because the availability of several broad spectrum anthelmintics is incorrectly believed to have reduced these infections in dogs and therefore their importance to animal and public health [1]. However, infections with intestinal nematodes (e.g. ascarids, ancylostomatids, trichurids) and cestodes are still very common and prevalent in dogs in Europe [2]. More attention has been paid in the last few years towards these “old-fashioned” parasites and many aspects of their biology, epidemiology, diagnosis and control have recently been reviewed [1,3,4]. Furthermore, infections with *Angiostrongylus vasorum* and other cardio-pulmonary nematodes are being increasingly diagnosed in dogs [5].

Kennel dogs are at the highest risk of infection with intestinal and extra-intestinal helminths. As an example, the findings of a recent survey in kennel dogs from southern Italy [6], despite the regular use of anthelmintic treatments, showed high prevalence of *Trichuris vulpis* (96.8%), *Toxocara canis* (74.2%), hookworms (67.7%), *Dipylidium caninum* (56.7%) and *Angiostrongylus vasorum* (11.3%).

Anthelmintics or combinations of anthelmintics with a broad spectrum activity are required to treat the polyparasitism currently encountered in kennel dogs. Various guidelines for the treatment and control of parasitic infections in companion animals have been proposed in the United States (by the Centers for Disease Control and Prevention, and the Companion Animal Parasitology Council - CAPC) and in Europe (by the European Scientific Counsel Companion Animal Parasitology - ESCCAP). The guidelines from ESCCAP recommend a careful consideration of helminth control programmes for dogs in kennels and a regular copromicroscopic monitoring to assess the effectiveness of control programmes (www.esccap.org).

Therefore, regular parasitological surveillance, appropriate treatment strategies and high quality standard of hygiene are required to guarantee the health and welfare of kennel dogs. The aim of the present study was to evaluate the efficacy of helminth control in kennels using different broad-spectrum anthelmintics that are routinely used in the canine veterinary practice. Particular attention was given to the field efficacy, palatability and easy-of-use of each product.

## Methods

### Study area and study kennels

The field trial was conducted between June and August 2013 in the Campania region of southern Italy (Latitude = 39°59′15″–41°30′25″; Longitude = 13°45′25″–15°48′23″) which extends over an area of 13,590 km^2^. The region is mainly hilly with an altitude between 0 and 1,890 m above sea level. The climate is Mediterranean with dry summers and winter rains. At the time of the study, 67 public kennels were present in the Campania region (www.anagrafecaninacampania.it). These kennels are facilities for roaming and abandoned animals; dogs are sheltered and then possibly adopted. The Italian regulation for dog registration/identification (Legge 281/1991) provides for setting up specific kennels to collect and re-home roaming and abandoned animals; for many disowned dogs the kennel becomes a permanent shelter [7]. As in other Italian regions, the kennels in Campania are subjected to veterinary public service control. The number of anthelmintic treatments given per year range from 1 (15%), 2 (75%) to 3 (10%) using broad-spectrum antiparasitic drugs (Cringoli, personal communication).

The study was conducted in 3 public kennels selected based on infection with at least 3 different helminth species. Informed consent was obtained from the kennels’ owners and veterinarians before enrolment in the study. The three kennels were located in plain areas, surrounded by intensively cultivated fields. Dogs were sheltered in boxes organized in different lines.

In these kennels, the dogs are fed once per day with standard commercial dog food at the recommended rates, while tap water is available *ad libitum*.

### Experimental design and selection of the epidemiological units (boxes)

In each of the three study kennels, the box (hosting a different number of dogs, i.e. min 1, max 4; mean = 2.4 dogs) was considered as the epidemiological unit of the study for practical reasons. Indeed, although careful attention was paid by the personnel collecting samples in order to guarantee that all the dogs in the same box were sampled, the precise identification of the dog which produced the faeces was not always possible during the entire study. This is the reason why we chose the box (i.e. the group of dogs using pooled faecal samples, see below) as epidemiological unit for our study rather than the single dog (using individual faecal samples).

At Day-35, boxes were selected in each kennel for faecal examination. Inclusion criteria were based on boxes with a minimum number of 2 dogs of any breed (provided they were non aggressive) and sex, aged more than 6 months. The number of boxes (and dogs) selected in each kennel is given in Table 1. Faecal exams were performed in each of the selected boxes at Day-30. The parasitological infections found in the three kennels under study (number of positive boxes for each helminth species) is given in Table 1. At Day −7, based on the coprological results obtained at Day −30 (i.e. geometric mean eggs per gram of faeces (epg) of either hookworms or ascarids or trichurids above 50 epg), 18 boxes were selected in each kennel for treatment. The group allocation and randomisation were conducted independently in each kennel following the same procedures in order to target the treatments towards 6 boxes positive for hookworms, 6 boxes positive for ascarids and 6 boxes positive for trichurids.

**Table 1:** **Parasitological scenario in the three kennels under study: number (no.) of boxes examined and no. of boxes positive for each helminth**

**Kennel**	**No. of boxes (dogs) selected and examined at Day −30 in each kennel**	**Hookworms**	***Toxocara canis***	***Trichuris vulpis***	***Dipylidium caninum***
1	100 (260) % (95% CI)	18 (48) 18.0% (11.3-27.2)	12 (30) 12.0% (6.6-20.4)	36 (90) 36.0% (26.8-46.2)	0
2	52 (164) % (95% CI)	9 (30) 17.3% (8.7-30.8)	8 (25) 15.4% (7.3-28.6)	28 (88) 53.8% (39.6-67.5)	5 (16) 9.6% (3.6-21.8)
3	98 (248) % (95% CI)	27 (69) 27.5% (19.2-37.6)	17 (44) 17.3% (10.7-26.6)	31 (80) 31.6% (22.8-41.9)	10 (26) 10.2% (5.3-18.4)
TOT	250 (672) % (95% CI)	54 (147) 21.6% (16.8-27.3)	37 (99) 14.8% (10.8-20.0)	95 (258) 38.0% (32.0-44.4)	15 (42) 6.0% (3.5-9.9)

### Treatments

The treatments (and efficacy studies) were conducted in 54 boxes in the three kennels (18 boxes per kennel; see Table 2) using tablets containing combinations of: i) milbemycin oxime (0.5 mg/kg) and praziquantel (5 mg/kg) (Milbemax®-chewable tablets, Novartis Animal Health, Inc.); ii) pyrantel embonate (14.4 mg/kg), febantel (15 mg/kg) and praziquantel (5 mg/kg) (Drontal Plus Flavour® tablets, Bayer); iii) pyrantel pamoate (5 mg/kg), oxantel pamoate (54 mg/kg) and praziquantel (5 mg/kg) (Nemex® POP tablets, Zoetis).

**Table 2:** **Treatment groups (no. of boxes and no. of dogs) used for the study**

**Treatment groups**	**No. of boxes (dogs) used for treatment in the three kennels**	**Hookworms**	***Toxocara canis***	***Trichuris vulpis***	***Dipylidium caninum***
Group T1	18 (51)	6 (18)	6 (18)	6 (15)	0
Group T2	18 (48)	6 (16)	6 (15)	6 (17)	6 (16)
Group T3	18 (47)	6 (14)	6 (16)	6 (17)	6 (16)
TOT	54 (146)	18 (48)	18 (49)	18 (49)	12 (32)

T1 = Group treated with Milbemax® (Novartis Animal Health, Inc.) tablet containing milbemycin oxime (0.5 mg/kg) and praziquantel (5 mg/kg); T2 = Group treated with Drontal Plus Flavour® (Bayer) tablet containing pyrantel embonate (14.4 mg/kg), febantel (15 mg/kg) and praziquantel (5 mg/kg); T3 = Group treated with Nemex® POP (Zoetis) tablet containing pyrantel pamoate (5 mg/kg), oxantel pamoate (54 mg/kg) and praziquantel (5 mg/kg).

The selected boxes were numbered, and a series of random numbers were generated by computer. Allocation of the dogs to Group T1 (to be treated with Milbemax®), Group T2 (to be treated with Drontal Plus Flavour®), Group T3 (to be treated with Nemex® POP) was randomized. At Day 0, all dogs in the boxes of the Groups T1, T2 and T3 were treated following the instructions for use of the corresponding product (Table 2). Dogs were kept (i.e. housing, food, etc.) under their usual housing conditions before, during and after the study.

No other products were used on the study animals or in their environment during the study. The study was conducted according to the VICH guidelines 9 [8–10] and the WAAVP guideline for evaluating the efficacy of anthelmintics for dogs and cats [11]. The study protocol was approved by the Animal Care Committee at the Department of Veterinary Medicine and Animal Productions, University of Naples Federico II (protocol number 456/2013).

### Faecal exams

Faecal exams were performed at Day −30, Day 0, Day 7, Day 14 and Day 21. At each sampling time, fresh faecal samples were collected from the ground of each box (pooled samples), transported to the laboratory (within 5 hours from sampling) and fixed in 5% formalin [12]. The average time from collection to egg counting was 6 days (range: 1–10 days). Copromicroscopic exams were performed using the FLOTAC dual technique [12], which is based upon the use of two flotation solutions that are used in parallel on the same faecal sample. A sodium chloride based solution (specific gravity –s.g. = 1.200) was used in order to detect nematode and cestode eggs [12,13], and a zinc sulphate based solution (s.g. = 1.200) was used to detect lungworm larvae [14,15] (data not shown). The analytic sensitivity of the FLOTAC dual technique was 2 eggs per gram (epg) of faeces. The kennel and laboratory staff conducting the copromicroscopic exams were blinded to the dog treatments.

At each sampling time after treatment (Day 7, Day 14 and Day 21), clinical observations were recorded and any adverse event individually registered in accordance with the VICH GL9 (GCP) rules [8].

### Data analysis

Efficacy of the treatments were assessed for each helminth detected in each study kennel. Efficacy against nematodes (hookworms, *T.canis* and *T.vulpis*) was assessed by analysing the reduction in faecal egg counts (FECR) between the first faecal sample (Day 0) and the three faecal samples obtained post treatment (Days 7, 14 and 21). Efficacy (% reduction in FEC) was calculated according to the following formula [2]:

$$ \%\ \mathrm{Reduction}=\left(\mathrm{N}1\ \hbox{--}\ \mathrm{N}2\right)/\mathrm{N}1 \times 100 $$

where, N1 was the geometric mean (GM) faecal egg count before treatment (Day 0) and N2 was the GM faecal egg count after treatment (Day 7, Day 14, Day 21).

A treatment was considered effective if a reduction in FEC of 90% or more was found (www.esccap.org). The results were analyzed by a non-parametric paired test (Wilcoxon-signed rank test) to evaluate the differences in epg before and after treatment (the significance level was set at 5%).

The efficacy against cestodes (*D.caninum*) was assessed as presence/absence [2]. Specifically, efficacy was measured by the proportion of boxes positive for cestodes (demonstrated by FEC and/or proglottids) at Day 0 that were negative for cestode eggs/proglottids at Days 7, 14 and 21.

## Results

Two hundred and fifty epidemiological units (boxes) of the three kennels under study complied with the inclusion criteria (dogs not aggressive of any breed or sex, aged above 6 years) and helminth infections were identified at Day −30 (Table 2).

Intestinal nematodes (hookworms, *Toxocara* and *Trichuris*) were present in all the three kennels; cestodes (*Dypilidium*) in 2/3 kennels. *T. vulpis* was the most prevalent species, found in 95 out of the 250 (38.0%; 95% CI = 32.0-44.0) boxes examined, followed by hookworms (54/250; 21.6%; 95% CI = 16.8-27.3), *T. canis* (37/250; 14.8%; 95% CI = 10.8-20.0) and *D. caninum* (15/250; 6.0%; 95% CI = 3.5-9.9).

Dogs of different genders (male, female, neutered male, spayed female), breeds, size (small, medium, large) and age (from 6 months to 13 years) were included in the treatments.

The efficacy (% reduction of GM faecal egg counts) of treatments against hookworms, *T. canis* and *T. vulpis* are given in Tables 3, 4 and 5 for kennel no. 1, 2 and 3, respectively.

**Table 3:** **Kennel 1: Geometric mean (GM), range and percentage reduction of faecal egg counts (epg) of nematode parasites in the 3 treatment groups**

**Treatment**	**Hookworms**				***Toxocara canis***				***Trichuris vulpis***			
**Group**												
	**Day 0**	**Day 7**	**Day 14**	**Day 21**	**Day 0**	**Day 7**	**Day 14**	**Day 21**	**Day 0**	**Day 7**	**Day 14**	**Day 21**
T1 (GM epg)	86.2	0	0	0	223.4	0	0	0	132.1	0	0	0
Range (epg)	2-190	0	0	0	28-1564	0	0	0	24-1010	0	0	0
***% reduction***	***-***	***100***	***100***	***100***	***-***	***100***	***100***	***100***	***-***	***100***	***100***	***100***
T2 (GM epg)	76.3	0	0	0	245.8	0	0	0	129.5	2.4	0	0
Range (epg)	2-180	0	0	0	8-2126	0	0	0	24-722	0-8	0	0
***% reduction***	***-***	***100***	***100***	***100***	***-***	***100***	***100***	***100***	***-***	***98.1***	***100***	***100***
T3 (GM epg)	82.5	0	0	0	254.2	8.2	0	0	163.3	4.3	0	0
Range (epg)	8-136	0	0	0	62-892	0-28	0	0	2-892	0-28	0	0
***% reduction***	***-***	***100***	***100***	***100***	***-***	***96.7***	***100***	***100***	***-***	***97.3***	***100***	***100***

T1 = Group treated with Milbemax® (Novartis Animal Health, Inc.); T2 = Group treated with Drontal Plus Flavour® (Bayer); T3 = Group treated with Nemex® POP (Zoetis).

**Table 4:** **Kennel 2: Geometric mean (GM), range and percentage reduction of faecal egg counts (epg) of nematode parasites in the 3 treatment groups**

**Treatment**	**Hookworms**				***Toxocara canis***				***Trichuris vulpis***			
**Group**												
	**Day 0**	**Day 7**	**Day 14**	**Day 21**	**Day 0**	**Day 7**	**Day 14**	**Day 21**	**Day 0**	**Day 7**	**Day 14**	**Day 21**
T1 (GM epg)	65.5	0	0	0	88.7	0	0	0	146.3	0	0	0
Range (epg)	2-534	0	0	0	2-422	0	0	0	2-932	0	0	0
***% reduction***	**-**	***100***	***100***	***100***	**-**	***100***	***100***	***100***	**-**	***100***	***100***	***100***
T2 (GM epg)	76.3	0	0	0	75.8	2.3	0	0	138.5	0	0	0
Range (epg)	36-588	0	0	0	2-922	0-4	0	0	48-672	0	0	0
***% reduction***	**-**	***100***	***100***	***100***	***-***	***97.1***	***100***	***100***	***-***	***100***	***100***	***100***
T3 (GM epg)	80.6	0	0	0	92.9	4.0	0	0	112.9	4.0	5.2	0
Range (epg)	2-626	0	0	0	18-1080	0-18	0	0	4-526	0-8	0-10	0
***% reduction***	**-**	***100***	***100***	***100***	**-**	***95.7***	***100***	***100***	**-**	***96.4***	***95.4***	***100***

T1 = Group treated with Milbemax® (Novartis Animal Health, Inc.); T2 = Group treated with Drontal Plus Flavour® (Bayer); T3 = Group treated with Nemex® POP (Zoetis).

**Table 5:** **Kennel 3: Geometric mean (GM), range and percentage reduction of faecal egg counts (epg) of nematode parasites in the 3 treatment groups**

**Treatment**	**Hookworms**				***Toxocara canis***				***Trichuris vulpis***			
**Group**												
	**Day 0**	**Day 7**	**Day 14**	**Day 21**	**Day 0**	**Day 7**	**Day 14**	**Day 21**	**Day 0**	**Day 7**	**Day 14**	**Day 21**
T1 (GM epg)	108.7	0	0	0	142.7	0	0	0	142.1	0	0	0
Range (epg)	2-674	0	0	0	20-682	0	0	0	28-242	0	0	0
***% reduction***	**-**	***100***	***100***	***100***	**-**	***100***	***100***	***100***	**-**	***100***	***100***	***100***
T2 (GM epg)	123.4	0	0	0	156.2	0	0	0	121.7	5.4	4.1	0
Range (epg)	2-428	0	0	0	2-624	0	0	0	2-530	0-30	0-10	0
***% reduction***	**-**	***100***	***100***	***100***	**-**	***100***	***100***	***100***	**-**	***95.6***	***96.6***	***100***
T3 (GM epg)	99.7	0	0	0	163.7	6.1	0	0	132.0	6.5	5.6	0
Range (epg)	42-282	0	0	0	18-954	2-24	0	0	2-629	0-56	0-30	0
***% reduction***	**-**	***100***	***100***	***100***	**-**	***96.3***	***100***	***100***	**-**	***95.1***	***95.7***	***100***

T1 = Group treated with Milbemax® (Novartis Animal Health, Inc.); T2 = Group treated with Drontal Plus Flavour® (Bayer); T3 = Group treated with Nemex® POP (Zoetis).

Very high efficacy (% reduction in GM epg) was obtained against hookworms, *T. vulpis* and *T. canis* at the different times with all anthelmintics tested. The differences in epg before and after treatments were highly significant (P <0.001) for each helminth at each sampling time and for each antiparasitic drug.

Specifically, Milbemax® showed 100% efficacy against all the helminthes in all the kennels and at each sampling time.

The efficacy of Drontal Plus Flavour® was 100% against hookworms in all the kennels; however, at some sampling times the efficacy was lower than 100% against *T. canis* (97.1% at Day 7 in kennel no. 2) and *T. vulpis* (98.1% at Day 7 in kennel no. 1; 95.6% and 96.6% at Days 7 and 14, respectively, in kennel no. 3).

The efficacy of Nemex® POP was 100% against hookworms in all the kennels; however, at some sampling times the efficacy was lower than 100% against *T. canis* (96.7% at Day 7 in kennel no. 1; 95.7% at Day 7 in kennel no. 2; 96.3% at Day 7 in kennel no. 3) and *T. vulpis* (97.3% at Day 7 in kennel no. 1; 96.4 and 95.4% at Days 7 and 14, respectively, in kennel no. 2; 95.1% and 95.7% at Days 7 and 14, respectively, in kennel no. 3).

In kennels no. 2 and no. 3, the boxes positive for cestodes (*D. caninum*) at Day 0 were negative at Days 7, 14 and 21 after treatments with either Milbemax® or Drontal Plus Flavour® or Nemex® POP (efficacy = 100%).

It should be noted that the differences between the efficacies of the three antiparasitic combinations were not statistically significant (P >0.05).

No adverse reaction to any treatment was observed during the study thus confirming the safety of the broad-spectrum anthelmintics used in the present study.

## Discussion and conclusions

Significant findings emerged from the present study regarding: (i) polyparasitism in kenneled dogs; (ii) the use of pooled faecal samples and FLOTAC for assessment of helminth infection (FEC) and thus anthelmintic drug efficacy (FECR); and (iii) the efficacy and applicability of Milbemax® compared to other broad-spectrum anthelmintics in kennels under field conditions.

First, the results of the present study confirmed that polyparasitism is the norm in kenneled dogs; the parasitological infections found in the present study were similar to worm infections of dogs in Europe [2]. Intestinal nematodes (with high prevalence of *Trichuris*, followed by hookworms and *Toxocara*) were found in the three kennels under study, whereas cestodes (*Dypilidium*) were less prevalent. Intestinal helminths (hookworms, ascarids, trichurids and cestodes) of dogs can cause anorexia, anemia and diarrhea associated with considerable morbidity and mortality rates, particularly in young, old and immune-compromised animals [16] which are the categories of dogs usually sheltered in kennels worldwide. This situation may be further exacerbated by the presence of cardio-pulmonary parasites such as *A. vasorum*, a helminth increasingly diagnosed in dogs in Europe and beyond [17].

Second, in the present study the assessments of helminth infection (FEC) and anthelmintic drug efficacy (FECR) were performed on pooled faecal samples (considering the boxes in the kennels as the epidemiological units) and using the very highly sensitive FLOTAC dual technique [12,18]. Pooled FEC/FECR, in which equal amounts of faeces from different individuals (i.e. dogs in the boxes of the kennels in the present study) are mixed together, is now considered a rapid procedure that holds promise as a valid strategy for FEC/FECR in the veterinary field [5,18]. The use of pooled FEC/FECR combined with the availability of a sensitive and multivalent technique such as FLOTAC might overcome some of the limits (e.g. time and cost to conduct sensitive and reliable copromicroscopic diagnosis) of the classical parasitological methods commonly used in the veterinary canine practice [13].

Third, with respect to the efficacy results, Milbemax® showed 100% efficacy against all the intestinal helminths (hookworms, *Trichuris, Toxocara* and *Dipylidium*) in all the kennels and at each sampling time (Days 7, 14 and 21). The results of our study are in line with the findings by Altreuther et al. [19] who reported high efficacy values of milbemycin oxime plus praziquantel tables against *T. vulpis* (99.6%), *T. canis* (99.8%), *T. leonina* (100%), *Ancylostoma caninum* (99.4%) and *D. caninum* (100%) in a multicenter field study.

The other broad-spectrum anthelmintics (Drontal Plus Flavour® and Nemex® POP) used in the present study also showed high values of efficacy against intestinal nematodes and cestodes confirming the findings of previous studies [20]. However, at some sampling times the efficacy was lower than 100% against *T. canis* and *T. vulpis*. Nevertheless, the differences between the efficacies of the three antiparasitic combinations were not statistically significant (P >0.05).

Broad-spectrum activity is an important feature of anthelmintic products to be used in dogs [19]. The advantages of an oral application of Milbemax® are additionally increased by the large spectrum of activity of praziquantel and milbemycin oxime against cestodes and nematodes (including cardio-pulmonary parasites) infecting dogs. It is highly efficacious to simultaneously treat and control hookworms, ascarids, trichurids and cestodes [19], but also to prevent cardiopulmonary dirofilariosis, and to reduce the level of infection of angiostrongylosis [21]. A recent study by Conboy et al. [22] showed that a single dose of Milbemax® was highly effective (98.7%) for the treatment of patent *Crenosoma vulpis* infection in dogs, a lungworm prevalent in southern Italy [23]. A high therapeutic efficacy (96.8%) of Milbemax® was also recently reported against the eyeworm *Thelazia callipaeda* in naturally infected dogs [24].

The use of a high sensitive technique such as FLOTAC confirmed the high efficacy of the tested drugs and the absence of anthelmintic resistance in kennels in southern Italy. The constant presence of parasitic infections in kennel dogs and the reoccurrence of them despite regular use of anthelmintics is probably due to constant re-infection rather than problems of inefficacies of drugs [25,26]. Therefore, it is clear that targeted anthelmintic treatments in kenneled dogs are strongly advised. ESCCAP guidelines should be considered when planning anthelmintic control programs in kennels with a minimum number of 4 administrations per year suggested (www.esccap.org).

No adverse reactions to any treatment were observed during the study also confirming the safety of Milbemax® and the other broad-spectrum anthelmintics used despite treating dogs from a wide range of ages, body weights and breeds.

In addition to efficacy and safety, the choice of an anthelmintic may be influenced by other features as, for example, ease of administration [3]; this aspect is particularly important in kennels where many dogs need to be treated at the same time. Indeed, a key point for controlling dog parasites is the lifelong chemopreventative program and broad-spectrum formulations with an easy mode of administration (e.g. chewy tablets) fit particularly with year-long worm control programs [4]. Furthermore, milbemycin oxime/praziquantel can be administered safely at a different time or for the whole pregnancy or lactation [1]. The possibility of anthelmintic treatments using molecules which can be administered safely during pregnancy and lactation is a point of pivotal importance when choosing antiparasitic drugs in kennels. As an example, Drontal ® Plus Flavour tablets for dogs have not been tested in the early stages of pregnancy, therefore, should not be used during the first two thirds of gestation.

It should be noted that in the present study Milbemax® and Drontal Plus Flavour®, unlike Nemex® POP, showed high acceptability in all the treated dogs thus confirming the findings by Altreuther et al. [19]. Broad-spectrum formulations with an easy mode of administration, i.e. chewy tablets characterized by tasty flavor, allow a treatment with minimal distress to the dogs [1] especially in a kennel setting. In addition, the ease-of-use of Milbemax® is increased by the fact that one tablet covers a wide range of weights of a dog (5–25 Kg), and therefore there is no need to break up the tablets as when using Drontal Plus Flavour® and Nemex® POP (one tablet for every 10 kg of bodyweight).

Based on the results of our study, milbemycin oxime plus praziquantel tablets are efficacious and convenient for the treatment and prevention of helminth infections in kennel dogs under field conditions.

## Abbreviations

CAPC: Companion Animal Parasitology Counsel
ESCCAP: European Scientific Counsel Companion Animal Parasitology
epg: eggs per gram of faeces
FEC: Faecal Egg Count
FECR: Reduction in Faecal Egg Counts
GM: Geometric mean
